# Bariatric Surgery Causing Hyperammonemia

**DOI:** 10.7759/cureus.5098

**Published:** 2019-07-08

**Authors:** Prathik Krishnan, Poornima Ramadas, David Landsberg

**Affiliations:** 1 Pulmonary Critical Care, State University of New York Upstate Medical University, Syracuse, USA; 2 Hematology Oncology, State University of New York Upstate Medical University, Syracuse, USA; 3 Internal Medicine, Crouse Hospital, Syracuse, USA

**Keywords:** bariatric surgery, nutritional deficiencies following bariatric surgery, hyperammonemia

## Abstract

Bariatric surgery is recognized as a highly effective therapy for obesity but it does carry a risk of short term and long term complications since it results in a permanent alteration of the patient's anatomy. We present a case of 45-year-old female presented with a macular rash on extremities and facial rash from a rehabilitation center after having been discharged a month earlier from a revision surgery on her gastric bypass for anastomotic bleeding. She progressively became lethargic with Magnetic Resonance Imaging (MRI) of the brain showed symmetrically restricted diffusion concerning for hypoxic injury. Her ammonia levels were at 142 micromoles per liter (mmol/L) at the initial check which worsened to 432 mmol/L over a few days, despite treatment. Laboratory investigation later revealed her to be deficient in zinc along with many essential and nonessential amino acids. Supplemental nutrition was initiated, specifically fortifying her parenteral feeds with the essential amino acid combinations that were found deficient on testing. This lead to a slow but progressive improvement in encephalopathy. This case highlights the importance of understanding the short and long term complications of bariatric surgery. Although neurological complications are rare with peripheral neuropathy being the most common one, hyperammonemic encephalopathy is a very severe complication, with incompletely understood mechanisms and predispositions, frequently resulting in failure of recognition and subsequent delays in intervention.

## Introduction

Roux-En-Y bariatric surgery is recognized as a highly effective therapy for obesity since it accomplishes a reduction in obesity with sustained weight loss and improvement in the quality of life. Bariatric surgery is one of the fastest growing fields in surgery and rapidly becoming the most common elective surgery. Like with all surgeries, bariatric surgery does carry the risk of short term and long term complications since it results in a permanent alteration of the patient's anatomy and causes malabsorption. We report a rare complication of hyperammonemia in a patient who had bariatric surgery (Roux-En-Y) caused by nutritional deficiency. 

## Case presentation

A 45-year-old female presented with macular extremity and facial rash from a rehabilitation center after having been discharged a month earlier from a revision surgery on her gastric bypass for anastomotic bleeding. The gastric surgery itself had been done four years prior to the presentation. On this admission she was noted to be lethargic for the past few days with neurological assessment revealing a Glasgow Coma Scale [1] score at 4, necessitating intubation and mechanical ventilation.

**Figure 1:**
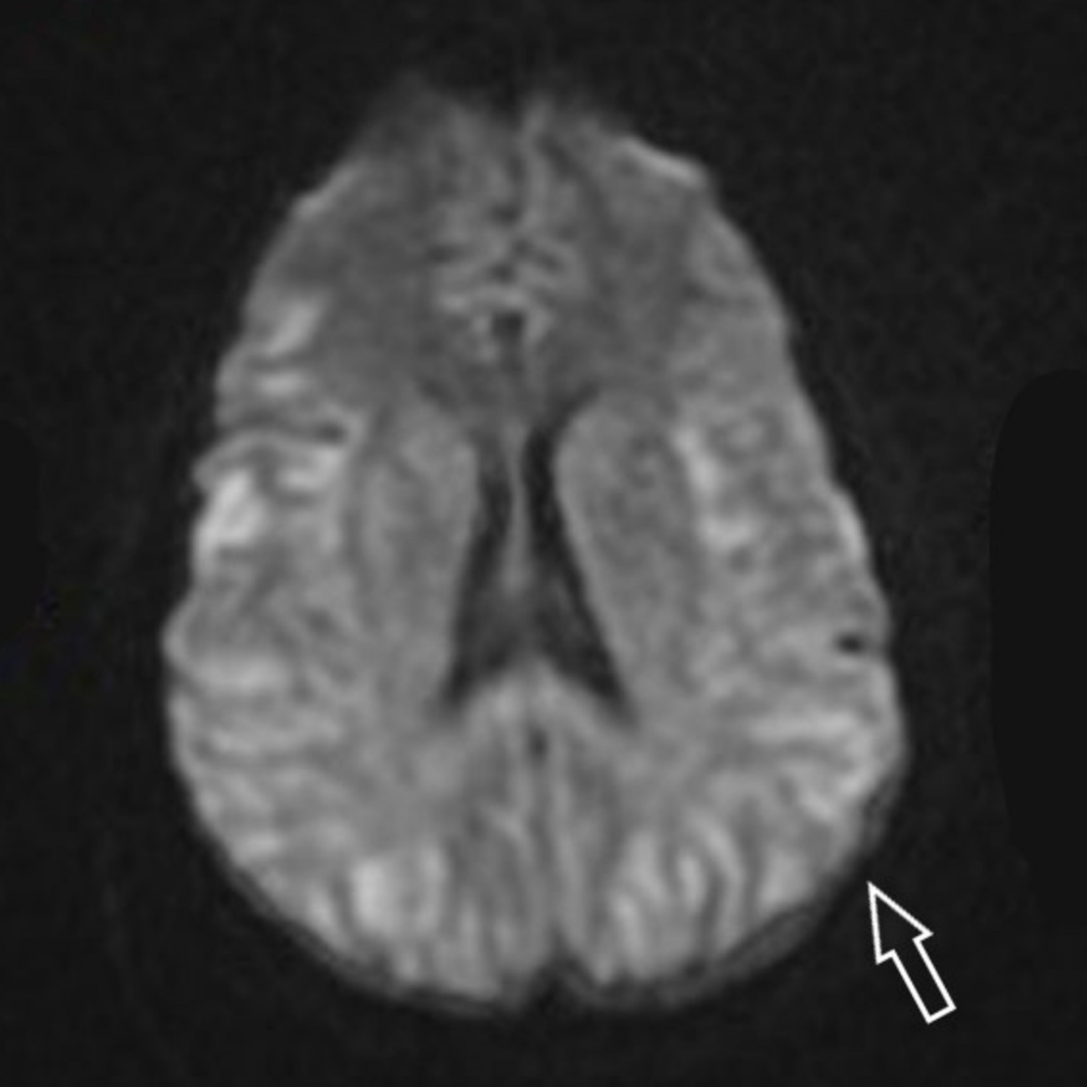
Magnetic Resonance Imaging of the brain showing symmetrically restricted diffusion Arrow over left parietal lobe showing areas of hyperintensity suspicious for hypoxic injury

During this time, extensive investigations were done including imaging of the head. The Magnetic Resonance Imaging (MRI) of the brain showed symmetrically restricted diffusion (Figure 1) was read as suspicious for hypoxic injury. This stood in contrast to any clinically documented hypoperfusion. Routine blood investigations did not offer a significant clue as her electrolytes, hepatic and renal functions were well within normal limits and the only thing that was seen abnormal was normocytic anemia, which in comparison to her past admissions, was found to be stable without an acute drop. Her thyroid profile, cortisol level, vitamin B12, and ammonia levels were checked for completion of the neurological workup and she was found to have elevated ammonia at 142 micromoles per liter (mmol/L) at the initial check. In the next few days of the critical care admission, she had progressive hyperammonemia to 432 mmol/L, despite treatment with lactulose and rifaximin. Ultrasound exams of the liver done during two different stages of the admission revealed no nodularity or fibrosis.

**Figure 2:**
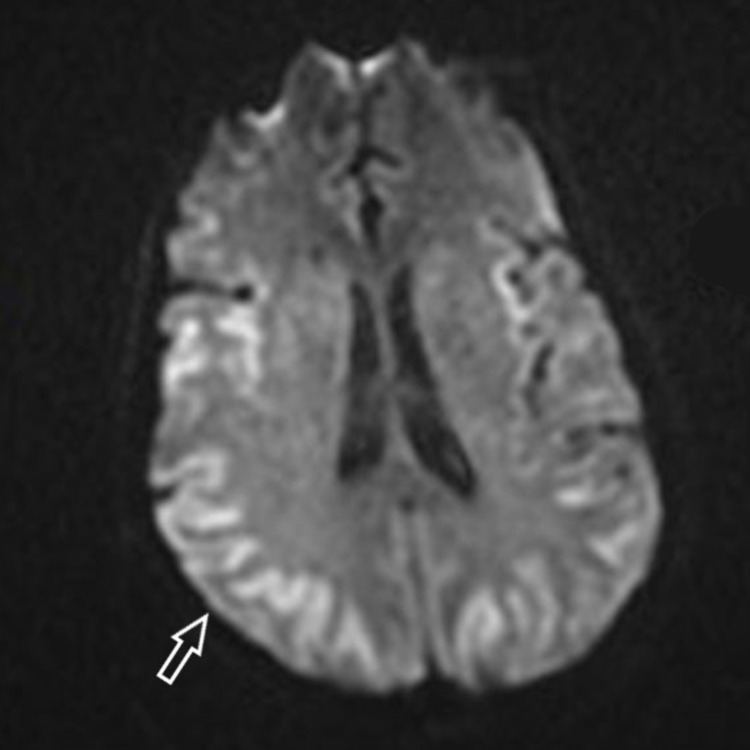
Magnetic Resonance Imaging of the brain showing worsening diffusion weighted abnormalities Arrow over right parietal lobe showing areas of hyperintensity suspicious for hypoxic injury

Continuous Renal Replacement Therapy (CRRT) was initiated despite normal renal function with the express intent to clear the ammonia and observe for improvement in encephalopathy while awaiting the results of extensive laboratory testing including serum levels of amion acids, essential minerals and vitamins to reveal the etiology. MRI (Figure 2) was repeated at that time showing worsening of diffusion weighted abnormalities. Laboratory investigation then revealed her to be deficient in zinc (Table 1) along with many essential and nonessential amino acids. The deficiencies are highlighted in (Table 2). On the third day of CRRT, supplemental nutrition was reinitiated, specifically fortifying her parenteral feeds with the essential amino acid combinations that were found deficient on testing.

**Table 1:** Serum levels of zinc

	Admission	Day 5	Day 24
Zinc (ug/dL) ref: 60-120	49	54	61

**Table 2:** Serum levels of amino acids

Amino acids	Detected (mmol/L)	Normal (mmol/L)
Arginine	34	40-160
Asparagine	29	30-80
Free Carnitine	24	25-60
Citrulline	9	10-60
Cystine	4	7-70
Glutamine	396	410-700
Histidine	44	50-110
Methionine	7	14-50
Phenylalanine	26	30-80
Serine	33	60-200
Taurine	9	25-150
Tryptophan	9	30-100
Tyrosine	7	30-120

**Figure 3:**
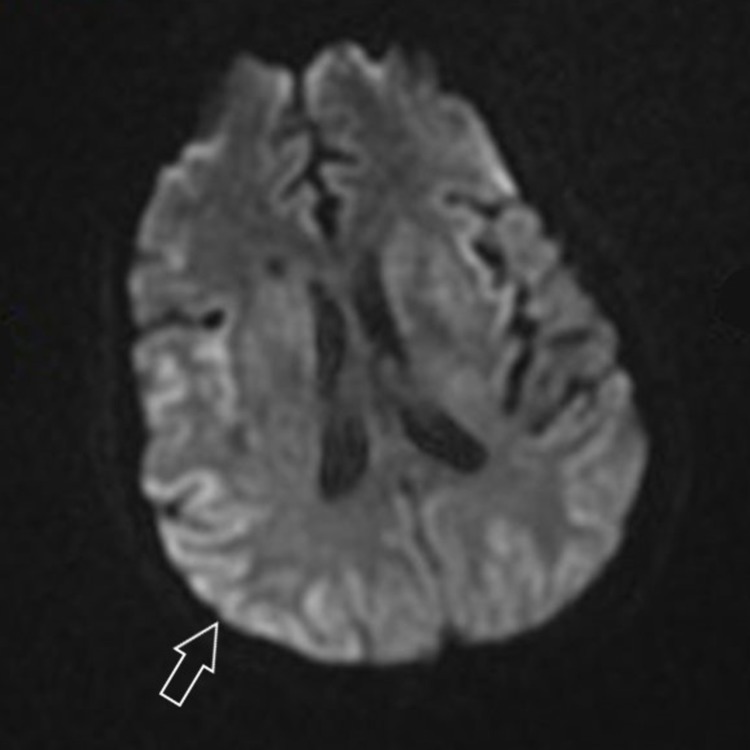
Magnetic Resonance Imaging of the brain showing reversal of diffusion weighted abnormalities Arrow over right parietal lobe showing areas of less hyperintensity denoting reversal of hypoxic injury

This lead to a change in her responsiveness, first to pain, then to loud voice commands in the couple of days to follow. A week later the patient was awake with what had been a slow but progressive improvement in encephalopathy. Repeat MRI (Figure 3) two weeks later showed substantial reversal of diffusion weighted changes and the patient herself was conversational and participating with physical therapy.

## Discussion

Ammonia, a toxic by-product of protein-energy metabolism, is converted to urea via the urea cycle and thereafter excreted through the kidneys. Abnormalities in this process can lead to hyperammonemia, which can be due to impaired hepatic metabolism, portal hypertension, encephalopathies of metabolic or toxic natures. Hyperammonemia can be toxic with signs and symptoms that include: episodic irritability, vomiting, ataxia, mental retardation, and lethargy that can progress to alteration of consciousness and coma. 

Both acute and chronic hyperammonemia alters the brain neurotransmitter system. Acute hyperammonemia causes accumulation of glutamate extracellularly in the brain, which activates the N-methyl D-aspartate receptor, causing seizures. Chronic hyperammonemia leads to an increase in inhibitory neurotransmission via down-regulation of glutamate receptors and increased GABAergic tone, causing deterioration of cognitive function and coma [2]. 

Multiple mechanisms have been hypothesized for the cause of hyperammonemia after gastric bypass surgeries. These include a significant reduction in the nutritive intake following the Roux-En-Y gastric bypass procedure may result in catabolism leading to protein breakdown in the extrahepatic tissues. Protein breakdown is typically followed by de‐amination and transamination of amino acids released from protein thus fueling hyperammonemia. Secondly, the possibility that gastric bypass surgery can interfere with the citrulline synthesis in the intestinal wall thereby leading to the depletion of urea cycle components and diminished ureagenic capacity culminating in hyperammonemia. Other mechanisms proposed include the gastric bypass surgery producing a blind gastric‐small bowel pouch, which may alter the gut microbiome favoring urealytic strains [3]. 

The management of hyperammonemia includes prevention of seizures and cerebral edema, medical therapy to remove excess ammonia and dietary protein restriction. Conservative management approaches with lactulose and rifaximin have resulted in modest reductions in measured ammonia levels. Repletion of deficient amino acids, zinc, micro nutrients, and intravenous glucose infusion may attenuate the catabolic state as seen in our patient. 

Obesity is a modern age burden and killer, with the Center for Disease Control and Prevention estimating that more than one-third (36.5%) of the adults in the United States have obesity [4]. Given that traditional prongs of weight loss including diet and lifestyle modifications are difficult to adhere to and are generally unsuccessful in the long run, bariatric surgery has made surges and is now one of the more common weight loss strategies in the United States, especially this century [5, 6]. However, despite extensive studies in the short and long term [7] safety and efficacy, a lot of complications are relatively less understood and require more studies. Neurologic complications are rarely seen and among those that have them, peripheral neuropathy is the most common [8]. However, an increasingly recognized entity, hyperammonemic encephalopathy is a very severe complication, with incompletely understood mechanisms and predispositions, frequently resulting in failure of recognition and subsequent delays in intervention.

## Conclusions

Roux-En-Y gastric bypass is the most common weight loss procedure performed in the United States and therefore this complication may be seen with increasing frequency. This case describes elevated ammonia as a complication of this surgery and the need for recognising this foreboding clinical presentation and its associated MRI findings, since it may readily respond to supplemental nutrition and normalise ammonia metabolism. 
